# Mutation Rates and Discriminating Power for 13 Rapidly-Mutating Y-STRs between Related and Unrelated Individuals

**DOI:** 10.1371/journal.pone.0165678

**Published:** 2016-11-01

**Authors:** Alessio Boattini, Stefania Sarno, Carla Bini, Valeria Pesci, Chiara Barbieri, Sara De Fanti, Andrea Quagliariello, Luca Pagani, Qasim Ayub, Gianmarco Ferri, Davide Pettener, Donata Luiselli, Susi Pelotti

**Affiliations:** 1 Dipartimento di Scienze Biologiche, Geologiche e Ambientali (BiGeA), Università di Bologna, Bologna, Italy; 2 Dipartimento di Scienze Mediche e Chirurgiche, Università di Bologna, Bologna, Italy; 3 Department of Archaeology and Anthropology, University of Cambridge, Cambridge, United Kingdom; 4 The Wellcome Trust Sanger Institute, The Wellcome Trust Genome Campus, Hinxton, United Kingdom; 5 Dipartimento di Medicina Diagnostica, Clinica e di Sanità Pubblica, Università degli Studi di Modena e Reggio Emilia, Modena, Italy; Harvard Medical School, UNITED STATES

## Abstract

Rapidly Mutating Y-STRs (RM Y-STRs) were recently introduced in forensics in order to increase the differentiation of Y-chromosomal profiles even in case of close relatives. We estimate RM Y-STRs mutation rates and their power to discriminate between related individuals by using samples extracted from a wide set of paternal pedigrees and by comparing RM Y-STRs results with those obtained from the Y-filer set. In addition, we tested the ability of RM Y-STRs to discriminate between unrelated individuals carrying the same Y-filer haplotype, using the haplogroup R-M269 (reportedly characterised by a strong resemblance in Y-STR profiles) as a case study. Our results, despite confirming the high mutability of RM Y-STRs, show significantly lower mutation rates than reference germline ones. Consequently, their power to discriminate between related individuals, despite being higher than the one of Y-filer, does not seem to improve significantly the performance of the latter. On the contrary, when considering R-M269 unrelated individuals, RM Y-STRs reveal significant discriminatory power and retain some phylogenetic signal, allowing the correct classification of individuals for some R-M269-derived sub-lineages. These results have important implications not only for forensics, but also for molecular anthropology, suggesting that RM Y-STRs are useful tools for exploring subtle genetic variability within Y-chromosomal haplogroups.

## Introduction

Small effective population size, male-specific inheritance and remarkable geographical differentiation make the human Y-chromosome one of the most used systems for exploring questions related to forensics and molecular anthropology [1–2]. Traditionally, Y-STRs are a valuable tool to differentiate male individuals belonging to different paternal lineages. The AmpFlSTR YFiler kit (Applied Biosystems, Foster City, CA, USA), which includes 17 highly variable Y-STR loci, has long represented the golden standard in most of the scientific work.

However, a number of criticisms have been raised, in particular concerning the susceptibility of STRs to homoplasy events [3–4]. This fact arises from the stepwise mutation model of STRs, according to which, when two consecutive mutations happen at the same locus, the latter will elide the former in half of the cases (backmutation). Consequently, identical (or very similar) Y-STR haplotypes may not be the result of a recent shared paternal ancestor, a well-known condition in forensic studies [5]. From a molecular anthropology point of view, homoplasic haplotypes would therefore mask the phylogenetic relationships between individuals. Y-chromosomal haplogroup R-M269 is one of the best known such examples: Y-STR haplotypes within this lineage typically show a strong resemblance even across different R-M269 sub-haplogroups. It has been hypothesized that such a behaviour may result from the combination of the recent, widespread radiation of R-M269 (which is the most frequent haplogroup in Western Europe) and the inherent tendency to homoplasy of STRs [4].

A second side effect of backmutations is saturation over long time scales [6]. This circumstance contributes to explain the uncertainty that hindered the choice of appropriate mutation rates for these markers: Y-STR mutation rates were estimated by direct count of mutation events in deep-rooting paternal pedigrees [7–9] or father-son pairs [5]. In contrast, 'evolutionary' rates were inferred from “populations with documented short-term histories” [10]. While methods based on direct count, in the case of the Yfiler set, yielded comparable results (pedigrees [9]: 3.90 * 10^−3^, father-son pairs [5]: 3.20 * 10^−3^), the evolutionary rate [10] is an order of magnitude lower (6.9 * 10^−4^). Such uncertainty on the choice of the “best mutation rate” affects all the analyses based on STR data, most notably the estimation of time of the most recent ancestor (TMRCA) of haplogroups (or, more in general, of groups of related haplotypes).

Recently, Ballantyne et al. [5] identified a set of Rapidly Mutating Y-chromosomal STRs (RM Y-STRs). Due to their exceptionally high mutability, the RM Y-STRs panel has proven abilities to substantially increase the differentiation of both related and unrelated males. In particular, these markers were reported to discriminate between fathers and sons in nearly 50% of cases, brothers in 60% and cousins in 75% [11], thus contributing to solve cases of homoplasy. In this respect, Larmuseau et al. [4] hypothesized that RM Y-STRs “will have a mutation rate too high to estimate the surname or designate the family of an unknown male”. However, their discriminating power in molecular anthropology studies is still untested. As for mutation rates, RM Y-STRs mutability is at the moment mainly documented by father-son comparisons [11–13].

In this study we explore the potential of 13 RM Y-STR markers for forensic and anthropological applications by newly estimating their mutation rates and testing their power to differentiate between related and unrelated individuals. Three different sets of data were analysed. The first two (Datasets A and B) include distantly and closely related individuals sampled within a wide set of deep-rooted paternal pedigrees; the third one (Dataset C) is made of unrelated individuals affiliated to nine different sub-lineages of Y-chromosomal haplogroup R-M269, which is characterised by high Y-STR resemblance [4]. Pedigree-based sets (Datasets A and B) were used to estimate STRs mutation rates as well as to assess and compare the ability of RM Y-STRs and Yfiler STRs to differentiate individuals related at various degrees. Instead, Dataset C was used to evaluate RM Y-STRs discriminating power between unrelated individuals who nonetheless show identical or almost-identical (one-step difference) Yfiler STRs profiles, as well as to test for their ability to retain phylogenetic signal.

## Materials and Methods

### Materials

#### Pedigree-based datasets (Datasets A and B)

On the whole, pedigree-based datasets include 99 individuals from two populations of Northern Italy, namely Partecipanza of S. Giovanni in Persiceto (Province of Bologna, Italy) and Pievequinta (Province of Forlì-Cesena, Italy). In both cases, samples were selected on the basis of their affiliation to deep-rooted paternal pedigrees. Partecipanza buccal swabs were collected from 43 distantly-related individuals belonging to 15 paternal pedigrees as detailed in Boattini et al. [9], to which we refer for further information. The 56 Pievequinta saliva samples were newly collected for this study using the Oragene-DNA Self Collection Kit OG-500. These samples were selected based on their correspondence with 14 paternal pedigrees out of the 240 genealogies which were available thanks to the long-standing activity of the cultural association “Amici della Pieve” from Pievequinta. These pedigrees refer to paternal lineages (surnames) that are widespread in the area comprised among the cities of Forlì, Cesena and Ravenna and were compiled after a thorough inspection of all the existing ecclesiastic records of the area. For each of these 14 pedigrees we sampled four individuals as follows: two couples of distantly related individuals, each couple being composed by close relatives (father-son, brothers, uncle-nephew, cousins). The only exception is pedigree G16, which is represented by a single couple of close relatives and two distantly-related individuals. A complete graphical description of the considered pedigrees is represented in S1 Fig. Overall, the above mentioned 99 samples (43 from Partecipanza and 56 from Pievequinta) were organised as follows.

Dataset A: Distantly-related individuals (Partecipanza and Pievequinta). This dataset includes 66 individuals who are related through deep-rooted pedigrees (S1 Table, S1 Fig).

Dataset B: Closely-related individuals (Pievequinta). This dataset comprises 27 couples of samples (54 individuals) who are as close as father-son, brothers, uncle-nephew, cousins. One sample per couple (i.e. 27) appears in both Datasets A and B (S2 Table, S1 Fig).

#### R-M269 Dataset (Dataset C)

Dataset C (S3 Table) comprises 86 samples extracted from the panel of Italian populations used in Boattini et al. [14] according to the following inclusion criteria: a) affiliation to the R-M269 haplogroup, which is represented by the following nine sub-lineages: R-M269*, R-U106, R-L48, R-P312, R-SRY2627, R-U152, R-L2, R-L20, R-L21 (a synthetic description of their phylogenetic relationships is represented in S2 Fig); b) identical (or one-mutational-step different) Yfiler STRs haplotypes with at least another one of the considered samples. Dataset C was arranged in a pairwise fashion, i.e. samples were organised in 67 couples, each of them comprising individuals with identical or one-mutational-step different Yfiler STRs profiles. As a consequence, each individual may take part to more than one couple. Couples from Dataset C were further separated in two groups: Dataset C1 (30 couples with both individuals affiliated to the same R-M269 sub-lineage) and Dataset C2 (37 couples composed by individuals affiliated to different R-M269 sub-lineages).

#### Sampling information

The collection of biological samples from adult, healthy individuals was performed during various sessions from 2008 to 2012 (Partecipanza) and from 2013 to 2014 (Pievequinta). For all subjects, a written informed consent was obtained. The Ethics Committees at the Azienda Ospedaliero-Universitaria Policlinico S. Orsola-Malpighi of Bologna and the Comitato di Bioetica dell’Università di Bologna approved all procedures. The samples were processed in a linked anonymized form, and the confidentiality of personal information for each participant to the study was assured.

### Laboratory methods

Whole genome DNA was extracted from the Oragene-DNA collection kits according to manufacturer’s recommendation (Purification of genomic DNA from the Oragene^®^ collection kits, DNA Genotek) and quantified by using the fluorimetric method Qubit^®^ dsDNA BR Assay Kit (Life Technologies).

The newly-collected samples from Pievequinta were genotyped for the 23 Y-STRs loci implemented in the PPY23 panel (Promega) (17 markers in Yfiler plus loci DYS481, DYS533, DYS549, DYS570, DYS576 and DYS643). However, in order to maintain compatibility with available literature data [14,9], all haplotype-based analyses were performed by considering the 17 markers matching the Yfiler panel.

All the samples from Partecipanza, Pievequinta and R1b-subsets were newly typed for the full set of 13 RM Y-STRs by using three multiplex PCR assays as described in Robino et al. [12].

PCR products were analyzed on an ABI PRISM^®^ 310 Genetic Analyzer and RM Y-STRs allele calling was performed with GeneMapper ID software v3.2 (Life Technologies) as described in Ballantyne et al. [15].

Newly-generated Y-chromosome data are comprised in S1–S3 Tables.

### Statistical Methods

#### Mutation number and mutation rates

Y-STR mutation rates for RM and Yfiler Y-STR markers were estimated by direct count in datasets A (deep pedigrees) and B (close relatives). As for RM, rates were calculated both by considering the whole panel (RM13) and by excluding its most variable markers, i.e. DYS399S1 and DYS403S1a (RM11). For each pedigree, we computed the number of mutation events (according to the maximum parsimony method) and the number of generations separating the considered haplotypes. Outlier pedigrees—that is pedigrees showing an outlier mutations/generations ratio—where excluded from calculations as possibly affected by non-paternity events. We identified outlier pedigrees by controlling for their mutations/generations ratios and iteratively applying Grubbs tests (function grubbs.test, outliers package [16], R software [17]).

The average mutation rates for Yfiler STRs and RM Y-STRs were calculated by dividing the total number of observed mutations for the total number of generations and for the number of considered STRs. Confidence intervals (95%) were estimated by calculating 2.5% and 97.5% quantiles in a binomial distribution with n = number of meioses (generations) and p = number of observed mutations / number of meioses. To allow maximal resolution, multi-copy STRs from both datasets (i.e. DYS385 in Yfiler and DYF399S1, DYF387S1, DYF404S1 and DYF403S1a in RM Y-STRs) were included in the calculations as separated loci; however, since mutation events were computed within pedigrees, it is unlikely that apparently identical configurations were actually belonging to different haplotypes.

Since it is highly improbable that multiple independent mutation events involved the very same STR locus in a relatively short amount of time, multi-step mutations within pedigrees (Datasets A and B) were considered as single events (for instance, 36–34 = 1 multi-step mutation). As for Dataset C, in which unrelated couples of haplotypes are compared, multi-step mutations were instead considered as independent events and counted accordingly (for instance, 36–34 = 2 independent mutations).

Diachronic changes in mutation rates were assessed by grouping the couples of individuals from Datasets A and B in bins, according to the number of meioses separating them (1–10, 11–20, 21–26). Mutation rates were calculated for Yfiler STRs, RM13 and RM11 using the above described procedure within the three considered intervals.

#### Discriminating power and phylogenetic signal

RM13 and RM11 discriminating power has been assessed by counting the average number of mutations separating couples of R-M269 individuals sharing identical (or one-mutational-step different) Yfiler profiles (Dataset C). Comparisons were performed considering two groups: Dataset C1 (couples sharing identical R-M269 sub-haplogroup) and Dataset C2 (couples belonging to different R-M269 sub-lineages). In addition, actual close relatives (Dataset B) were introduced as a third group. Bootstrapped confidence intervals (95%) were estimated by randomly re-sampling mutations in couples of individuals (1000 replications).

In order to assess the phylogenetic signal carried by the considered STRs, we performed Discriminant Analysis of Principal Components (DAPC [18–19]) on STR haplotypes from Dataset C, by considering their a-priori assignment to one of the 9 above mentioned R-M269 sub-lineages. In particular, we tested and compared three different STR panels: Yfiler, RM11 and Yfiler + RM11. Since Dataset C is composed by unrelated individuals, multi-copy STRs were excluded from the analyses. Membership probabilities for each individual to belong to a given R-M269 sub-haplogroup were calculated and results were represented by means of admixture-like plots. In addition, we calculated average membership probabilities (amp) per sub-lineage.

## Results

### Mutation Rates

**Fig 1:**
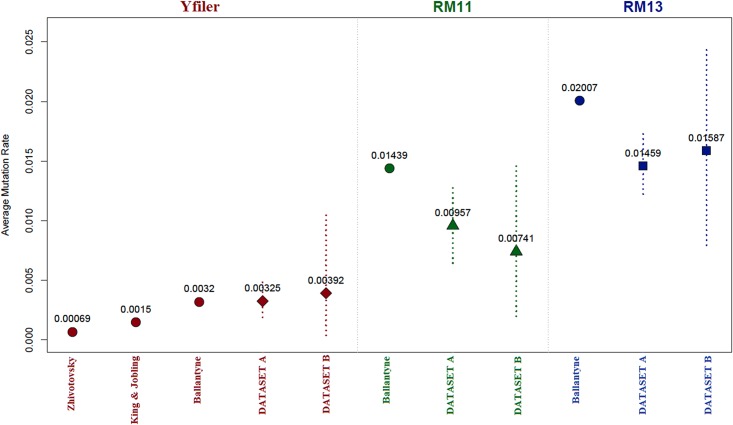
Average mutation rates for the considered Y-STR sets (Yfiler, RM11, RM13) in deep rooted pedigrees (Dataset A) and in close relatives (Dataset B). Dotted vertical lines represent 95% confidence intervals (when available). For comparison, estimates from literature are included^10,8,11^.

As a preliminary step, Grubb tests among the 29 pedigrees in Dataset A revealed three outliers (G19, G13 and G22). In all cases, these pedigrees show higher-than-expected mutations/generations ratios (pval = 7.13 * 10^−8^, pval = 6.02 * 10^−5^ and pval = 7.13 * 10^−10^, respectively). A further run of the test after having removed these pedigrees yielded a non-significant result (pval = 0.55), meaning that no more outliers were present in Dataset A. All further calculations were thus performed with the remaining 26 pedigrees and results are detailed in Table 1 and Fig 1.

**Table 1:** Mutation rates and 95% confidence intervals for the considered Y-STR sets (Yfiler, RM11, RM13) in pedigree-based Datasets (A and B). Diachronic changes in mutation rates were calculated for three increasing bins of generations (1–10, 11–20, 21–26). NGEN: total number of generations; NMUT: number of observed mutations.

DATASET	NGEN	NMUT	STR_SET	MUT_RATE	CI_2.5%	CI_97.5%
A	470	26	Yfiler	**0.00325**	0.00213	0.00451
A	470	96	RM13	**0.01459**	0.01201	0.01717
A	470	54	RM11	**0.00957**	0.00623	0.01033
B	45	3	Yfiler	**0.00392**	0.00000	0.00915
B	45	10	RM13	**0.01587**	0.00794	0.02540
B	45	4	RM11	**0.00741**	0.00185	0.01481
A + B (1–10)	168	8	Yfiler	**0.00280**	0.00105	0.00490
A + B (11–20)	447	23	Yfiler	**0.00303**	0.00184	0.00434
A + B (21–26)	630	34	Yfiler	**0.00317**	0.00215	0.00430
A + B (1–10)	168	34	RM13	**0.01446**	0.01020	0.01871
A + B (11–20)	447	87	RM13	**0.01390**	0.01135	0.01662
A + B (21–26)	630	109	RM13	**0.01236**	0.01032	0.01451
A + B (1–10)	168	17	RM11	**0.00843**	0.00496	0.01240
A + B (11–20)	447	58	RM11	**0.01081**	0.00820	0.01342
A + B (21–26)	630	76	RM11	**0.01005**	0.00794	0.01217

By considering 66 individuals within 26 pedigrees, 470 generations (meioses) were observed. Within them, 26 and 96 mutations for Yfiler and RM13 were detected, respectively. Considering the RM11 panel, the number of mutations is 54. Accordingly, Yfiler average mutation rate is 3.254 * 10^−3^ [CI: 2.128 * 10^−3^, 4.506 * 10^−3^]. As for RM Y-STRs, our pedigree-based estimations confirm their higher mutability with respect to Yfiler, yielding a rate equal to 1.46 * 10^−2^ [CI: 1.20 * 10^−2^, 1.73 * 10^−2^] for the full panel (RM13) and 9.57 * 10^−3^ [CI: 6.20 * 10^−3^, 1.27 * 10^−2^] after removing the most mutable loci (RM11).

Yfiler locus-by-locus mutation rates (Table 2) are significantly correlated to Ballantyne's et al. [11] estimates (Pearson’s r = 0.58, pval = 0.014). As for RM Y-STRs markers, the correlation is higher (Pearson’s r = 0.97, pval < 10^−8^) but our estimates are in all cases lower than those reported by Ballantyne et al. [11].

**Table 2:** Locus-by-locus mutation rates and 95% confidence intervals in Yfiler and RM Y-STRs base on deep-rooted pedigrees. For comparison, Ballantyne et al.^11^ estimates are reported (BALL). NMUT: number of observed mutations.

	NMUT	MUT_RATE	CI 2.5%	CI 97.5%	BALL
**Yfiler**					
DYS393	**2**	**0.00426**	0.00000	0.01064	0.00211
DYS390	**3**	**0.00638**	0.00000	0.01489	0.00152
DYS19	**2**	**0.00426**	0.00000	0.01064	0.00437
DYS391	**1**	**0.00213**	0.00000	0.00638	0.00323
DYS385a	**2**	**0.00426**	0.00000	0.01064	0.00208
DYS385b	**0**	**0.00000**	0.00000	0.00000	0.00414
DYS439	**1**	**0.00213**	0.00000	0.00638	0.00384
DYS389I	**1**	**0.00213**	0.00000	0.00638	0.00551
DYS392	**1**	**0.00213**	0.00000	0.00638	0.00097
DYS389II	**2**	**0.00426**	0.00000	0.01064	0.00383
DYS458	**6**	**0.01277**	0.00426	0.02340	0.00836
DYS437	**1**	**0.00213**	0.00000	0.00638	0.00153
DYS448	**0**	**0.00000**	0.00000	0.00000	0.00039
YGATA H4	**1**	**0.00213**	0.00000	0.00638	0.00322
DYS456	**1**	**0.00213**	0.00000	0.00638	0.00494
DYS438	**0**	**0.00000**	0.00000	0.00000	0.00096
DYS635	**2**	**0.00426**	0.00000	0.01064	0.00385
**RM Y-STRs**					
DYS576	**5**	**0.01064**	0.00213	0.02128	0.01430
DYF399S1	**30**	**0.06383**	0.04255	0.08723	0.07730
DYF387S1	**6**	**0.01277**	0.00426	0.02340	0.01590
DYS570	**2**	**0.00426**	0.00000	0.01064	0.01240
DYS526A+B	**3**	**0.00638**	0.00000	0.01489	0.01250
DYS626	**3**	**0.00638**	0.00000	0.01489	0.01220
DYS627	**9**	**0.01915**	0.00851	0.03191	0.01230
DYS518	**6**	**0.01277**	0.00426	0.02340	0.01840
DYS612	**5**	**0.01064**	0.00213	0.02128	0.01450
DYS449	**2**	**0.00426**	0.00000	0.01064	0.01220
DYS547	**7**	**0.01489**	0.00426	0.02766	0.02360
DYF404S1	**4**	**0.00851**	0.00213	0.01702	0.01250
DYF403S1a	**12**	**0.02553**	0.01277	0.04043	0.03100
DYF403S1b	**2**	**0.00426**	0.00000	0.01064	0.01190

In order to check if the discrepancies observed between RM Y-STRs germline rates and our estimates may be due to saturation (along deep pedigrees), we calculated mutation rates in close-relatives pairs (Dataset B). Results show that there is no significant difference between rates from Dataset A and Dataset B (Table 1, Fig 1).

Since Dataset B comprises a limited number of meioses/observations (hence large confidence intervals are yielded), we investigated diachronic changes in mutation rates by merging datasets A and B in 97 pairwise comparisons (within pedigrees), which are detailed in S4 Table. Mutation rates were calculated for three increasing bins of generations/meioses: 1–10, 11–20, 21–26.

**Fig 2:**
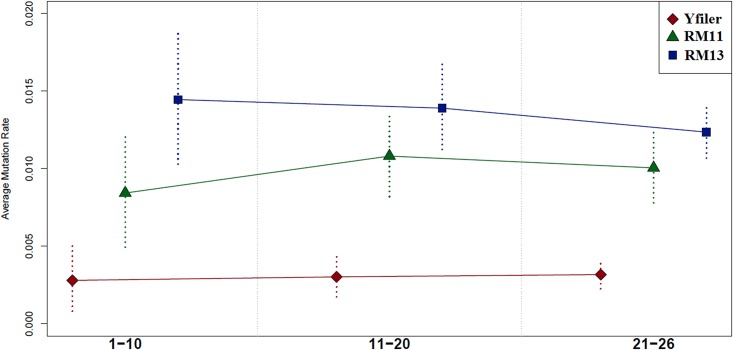
Diachronic changes in Yfiler, RM13 and RM11 average mutation rates based on three increasing bins of generations (1–10, 11–20, 21–26). Dotted vertical lines represent 95% confidence intervals.

Results (Table 1, Fig 2) confirm that Yfiler average rate does not change significantly with the number of generations/meioses, while RM13 rates tend to decrease with temporal depth. However, such trend is no more apparent when considering the RM11 panel. We conclude that the RM13 panel tendency to saturation is completely ascribable to its most variable markers (DYS399S1, DYS403S1a).

### Discriminating Power between close relatives

Among the 27 couples of closely related samples of Dataset B, only 2 of them show different Yfiler haplotypes, i.e. a discriminatory power of 7.41%. When shifting to RM Y-STRs panels, the records increase to 4 with RM11 and 9 with RM13, thus improving the discriminating capacity by two (14.82%) and four and a half (33.33%) times, respectively.

**Fig 3:**
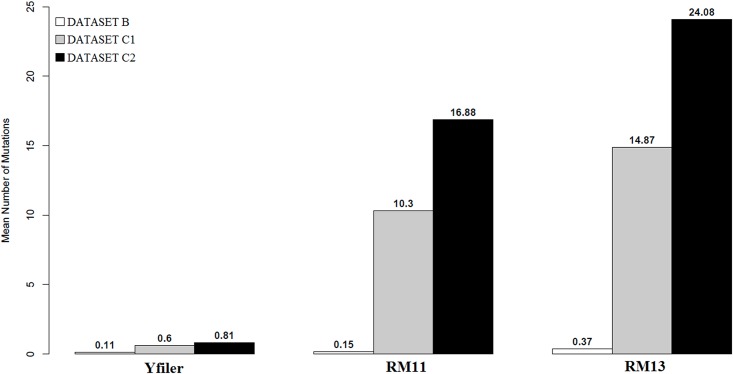
Mean number of observed mutations between individuals sharing identical (or one mutation different) Yfiler profiles affiliated to haplogroup R-M269. Dataset C1: couples of individuals affiliated to the same R-M269 sub-lineage; Dataset C2: couples of individuals affiliated to different R-M269 sub-lineages. Estimates for close relatives (Dataset B) are included.

As for the average number of observed mutations between couples in Dataset B, Yfiler averagely counts 0.11 mutations per couple [95% CI: 0.00, 0.30], RM11 0.15 [95% CI: 0.04, 0.30] and RM13 0.37 [95% CI: 0.18, 0.59] (S5 Table, Fig 3). Importantly, 95% CIs for the three sets largely overlap.

### Discriminating Power within haplogroup R-M269

When considering Dataset C (S5 Table, Fig 3), RM Y-STRs (RM11, RM13) yield significantly (pval = 3.2 * 10^−3^) higher values than those observed for close relatives (Dataset B), e.g. averagely 10.30 [95% CI: 7.73, 13.23] and 16.78 [95% CI: 15.22, 18.22] mutations for RM11 (Dataset C1 and Dataset C2, respectively) and averagely 14.87 [95% CI: 11.23, 18.77] and 24.08 [95% CI: 22.16, 25.97] for RM13 (Dataset C1 and Dataset C2, respectively). In all cases 95% CIs do not overlap with those calculated for Dataset B, meaning that both RM11 and RM13 discriminate neatly couples from Dataset C, clearly revealing their non-relatedness. Importantly, the mean number of mutations observed in Dataset C2 (different R-M269 sub-lineages) is significantly higher than in Dataset C1 (same R-M269 sub-lineage). We conclude that RM Y-STRs, despite their high mutability, still retain some phylogenetic signal.

### Discriminant Analysis between R-M269 sublineages

Accordingly, we explore to which extent RM Y-STRs and Yfiler Y-STRs correctly discriminate haplotypes among nine R-M269 sub-lineages (see Materials) by using DAPC.

**Fig 4:**
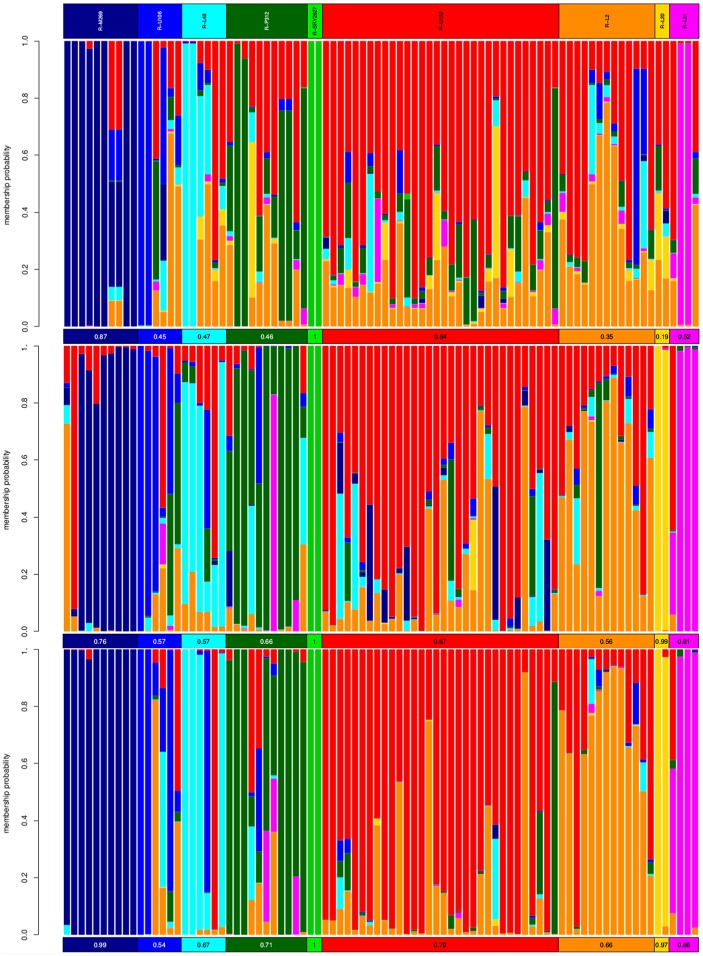
Discriminating power between R-M269 sub-lineages. Three STR panels are considered: Yfiler (top), RM11 (middle) and Yfiler + RM11 (bottom). Results are represented with admixture-like plots, in which vertical bars represent probabilities for each individual to be affiliated to a given R-M269 sub-lineage (membership probabilities). Mean per-group membership probabilities are reported in horizontal bars at the bottom of each plot.

Yfiler results (Fig 4) show that R-M269* samples are in most cases correctly assigned (amp = 0.87), while the other considered lineages are scarcely distinguishable, in particular U152-derived sub-haplogroups R-L20 (amp = 0.19) and R-L2 (amp = 0.35). Similarly, the second most differentiated sub-lineage (amp = 0.64) is R-U152* (i.e. excluding L2 and L20). As for RM11, our results show a slightly lower score for R-M269* (amp = 0.76) but a general improvement in all other cases, the most notable ones being R-L20 (amp = 0.99), R-L2 (amp = 0.56) and R-L21 (amp = 0.81). This suggests that RM yield an higher discriminatory power than Yfiler within R-M269, especially as far as the more derived lineages are concerned. By combining Yfiler and RM11 we observe a further improvement of the scores for all lineages (R-M269*: amp = 0.99; R-U152*: amp = 0.79). Nevertheless, some lineages still remain overall scarcely distinguishable, most notably R-U106 (amp = 0.54) and its derived lineage R-L48 (amp = 0.67).

## Discussion

Forensics and molecular anthropology share a set of common tools, among which Y-chromosome STRs are one of the most frequently used. In this study, we estimate mutation rates of two Y-STR sets—the well-known Yfiler kit and the newly-developed Rapidly Mutating 13 Y-STRs panel—by using deep-rooted paternal pedigrees, showing how they yield different discriminating power and phylogenetic signal.

As previously reported with a more restricted dataset [9], our estimate of Yfiler average mutation rate (3.254 * 10^−3^) matches perfectly with the germline rate calculated by Ballantyne et al. [5] (3.2 * 10^−3^). Both these values are higher than King and Jobling's [8] pedigree-based estimate and especially than the 'evolutionary' rate proposed by Zhivotovsky et al. [10], the latter being one order of magnitude slower (6.9 * 10^−4^). Interestingly, no saturation effects are apparent in the Yfiler markers (Table 1, Fig 1), at least within the time scale covered by our pedigrees.

As for RM Y-STR markers, our pedigree-based estimates confirm that they have a higher tendency to mutate than the Yfiler ones, in particular concerning the DYS399S1 and DYS403S1a loci, which show relatively high mutation rates (6.38 * 10^−2^ and 8.51 * 10^−2^, respectively; Table 2). This circumstance prompted us to perform analyses by using two different RM Y-STRs sets, i.e. RM13 (full set) and RM11 (after removing the two mentioned loci). When we compare our results with those by Ballantyne et al. [5,11], our pedigree-based estimates are significantly lower than germline rates (1.46 * 10^−2^ and 2.01 * 10^−2^ for RM13; 9.57 * 10^−3^ and 1.44 * 10^−2^ for RM11, respectively; Table 1, Fig 1), despite a general good correlation between the two sets of values (Table 2). In order to test whether such discrepancy may be somehow due to saturation effects within pedigrees, we calculated rates in three groups of pairs of related-individuals characterized by increasing number of generations. With the RM11 set, we did not find any appreciable difference (Table 1, Fig 2). On the other hand, when considering the full set (RM13), a decreasing trend (with the number of meioses/generations) was indeed found (Table 1, Fig 2). This fact shows that the highly mutable DYS399S1 and DYS403S1a loci are responsible of the observed trend, which in addition cannot be considered as statistically significant, since CIs largely overlap (Table 1, Fig 2). We then turned our attention to Dataset B, which includes pairs of closely related individuals. Even in this case, our estimates (Table 1) are similar to those obtained for the deep-rooted pedigrees (Dataset A), hence lower than Ballantyne’s et al. [11] germline rates. In summary, our results do not prompt any evidence of saturation in RM Y-STRs, with the partial (but not significant) exception of the two aforementioned multi-copy loci.

As for the discriminatory power of RM Y-STRs, Ballantyne et al. [11] reported that RM Y-STRs discriminate between fathers and sons in 50% of the cases and between brothers in 60%. Our results, are quite lower, with the full RM Y-STRs set (RM13) reaching a discriminatory power equal to 33.33%, while RM11 to 14.82% and YF to 7.41%.

The discriminatory power of Y-STRs sets can be also expressed in terms of average number of observed mutations (between couples of individuals). When considering close relatives (Dataset B), Yfiler and RM11 yield similar results, while RM13, due to the exceptionally high mutability of STRs DYS399S1 and DYS403S1a, outputs higher results; nevertheless their CIs largely overlap. In conclusion, at this stage we cannot state that RM—despite confirming their higher mutability—can actually represent a significant improvement in respect to Yfiler for discriminating close relatives.

On the contrary, RM Y-STRs revealed to be very efficient in cases of identical (or nearly identical) Yfiler profiles but actually belonging to unrelated individuals. Evidences of identical Yfiler profiles even between individuals which are differentiated by common SNPs are well documented, especially for haplogroup R-M269 [4]. We tested this by comparing RM Y-STRs haplotypes in couples of unrelated individuals within R-M269 showing identical (or one-mutation-step different) Yfiler profiles. Our results (Fig 3) attest the ability of RM Y-STRs (both RM11 and RM13) to actually discriminate these cases, with obvious consequences for the forensic work.

We investigated further by considering the nine R-M269 sub-lineages shared by our samples (Dataset C, see Materials). We observed that the average number of mutations detected by RM Y-STRs is significantly higher when couples are formed by individuals affiliated to different sub-haplogroups (Fig 3). We conclude that RM Y-STR markers not only do not seem affected by saturation, but also yield phylogenetic signal.

Such results have important implications for the molecular anthropology field. Can RM Y-STRs be used for phylogenetic ends, for example for grouping individuals according to their haplogroup? It has been shown that STR profiles may be used to infer the haplogroup of one individual [20,21]. As for RM, it was suggested that saturation would have cancelled any phylogenetic signal in a short amount of time [4]. In light of our results (Fig 4), within the relatively recent time-frame of haplogroup R-M269, Yfiler proves scarcely useful, since it works well only in distinguishing R-M269* from the other lineages. If RM Y-STRs yield a much better performance on the other sub-haplogroups, the combination of both datasets (Yfiler+RM) yields the best results. This result argues in favour of STRs as a still viable and cost-effective tool to investigate Y-chromosomal variation, moreover providing important insights into both forensic and molecular anthropology fields.

In conclusion, the principal outcomes of this study may be summarised as follows.

1. Yfiler average mutation rate based on our deep pedigrees (Dataset A) was shown to be quite similar to that reported by Ballantyne et al. [11], while RM Y-STRs revealed significantly lower values. Such difference cannot be related to saturation effects, at least within the time frame covered by our pedigrees.
2. Given their higher mutation rates, RM Y-STRs differentiate more efficiently even between tight relatives (Dataset B); nevertheless their discrimination power is not significantly higher than Yfiler one. Most importantly, the RM Y-STRs panel demonstrates significant discriminatory power in cases of Yfiler identical haplotypes within the widespread haplogroup R-M269 (Dataset C). These facts have important consequences from the forensics point of view, e.g. when paternal lineage differentiation or male relative separation are needed in casework [11].
3. As for the molecular anthropology field, our DAPC-based exploration of haplogroup R-M269 showed that RM Y-STRs improve significantly the performance of the method (with respect to Yfiler), allowing the correct classification of individuals at least for some R-M269-derived sub-lineages. We argue that Y-STR sets are a still valuable tool for both phylogenetic and phylogeographic inferences.

## Supporting Information

S1 Fig Paternal pedigrees included in the present study. Numbers along each branch represent the corresponding number of generations. For each pedigree is reported the total number of (NG). Color codes specify samples for Datasets A and B.
(PDF)

S2 Fig Phylogenetic relationships between the considered nine R-M269 lineages (R-M269*, R-U106, R-L48, R-P312, R-SRY2627, R-U152, R-L2, R-L20, R-L21). (TIF)

S1 Table Y-STR data (Yfiler, PPY23, RM) for 66 distantly-related individuals (Dataset A) from Partecipanza of S. Giovanni in Persiceto (PART) and Pievequinta (PQ). The corresponding paternal pedigrees are represented in S1 Fig. Yfiler data for Partecipanza were previously published in [9].
(XLS)

S2 Table Y-STR data (PPY23, RM) for 54 closely-related individuals (Dataset B) from Pievequinta (PQ). The corresponding paternal pedigrees are represented in S1 Fig.
(XLS)

S3 Table Y-STR data (Yfiler, RM) for 67 couples of individuals affiliated to nine sub-lineages of haplogroup R-M269 (Dataset C). Each couple comprises individuals with identical (or one-mutational-step different) Yfiler profiles and a) affiliation to the same R-M269 sub-lineage (Dataset C1) or b) affiliation to different R-M269 sub-lineages (Dataset C2). Yfiler information for this dataset was previously published in [14].
(XLS)

S4 Table Couples of related individuals used to investigate diachronic changes in mutation rates. Mutation rates were calculated for three increasing bins of generations/meioses: 1–10, 11–20, 21–26. For each couple, the number of generations/meioses (NGEN) and of observed mutations (NMUT) for each STR set (Yfiler, RM13, RM11) are reported.
(XLS)

S5 Table Average number of observed mutations between individuals sharing identical (or one mutation different) Yfiler profiles affiliated to haplogroup R-M269. Dataset C1: couples of individuals affiliated to the same R-M269 sub-lineage; Dataset C2: couples of individuals affiliated to different R-M269 sub-lineages. Estimates for close relatives (Dataset B) are included.
(XLS)
