# Supplementary material for: Mutation Rates and Discriminating Power for 13 Rapidly-Mutating Y-STRs between Related and Unrelated Individuals
Source: PLoS One. 2016 Nov 1;11(11):e0165678. doi: 10.1371/journal.pone.0165678 (PMC5089551; doi:10.1371/journal.pone.0165678)
Supplement: S1 Fig — Numbers along each branch represent the corresponding number of generations. For each pedigree is reported the total number of (NG). Color codes specify samples for Datasets A and B. (PDF) [file pone.0165678.s001.pdf]

PARTECIPANZA
(numbers along branches represent the number of generations; NG = total number of generations).

P01

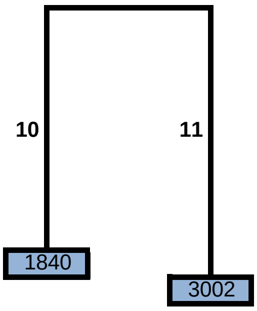

NG = 21

P02

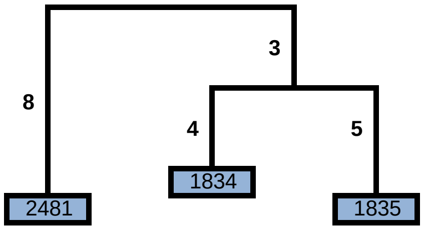

NG = 20

P03

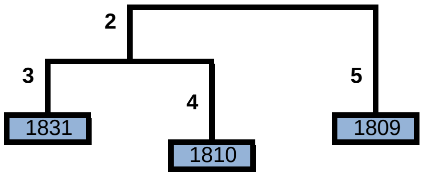

NG = 14

P05

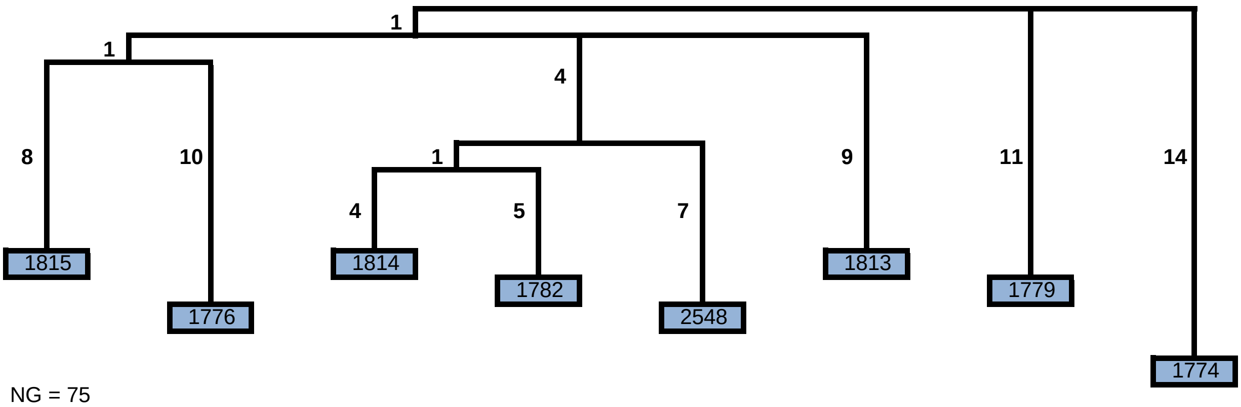

NG = 75

P04

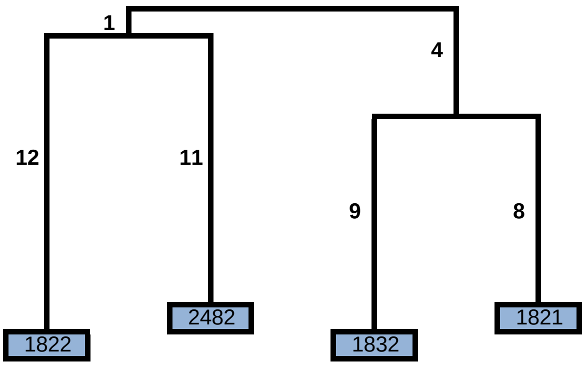

NG = 45

P06

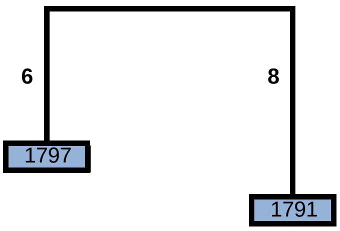

NG = 14

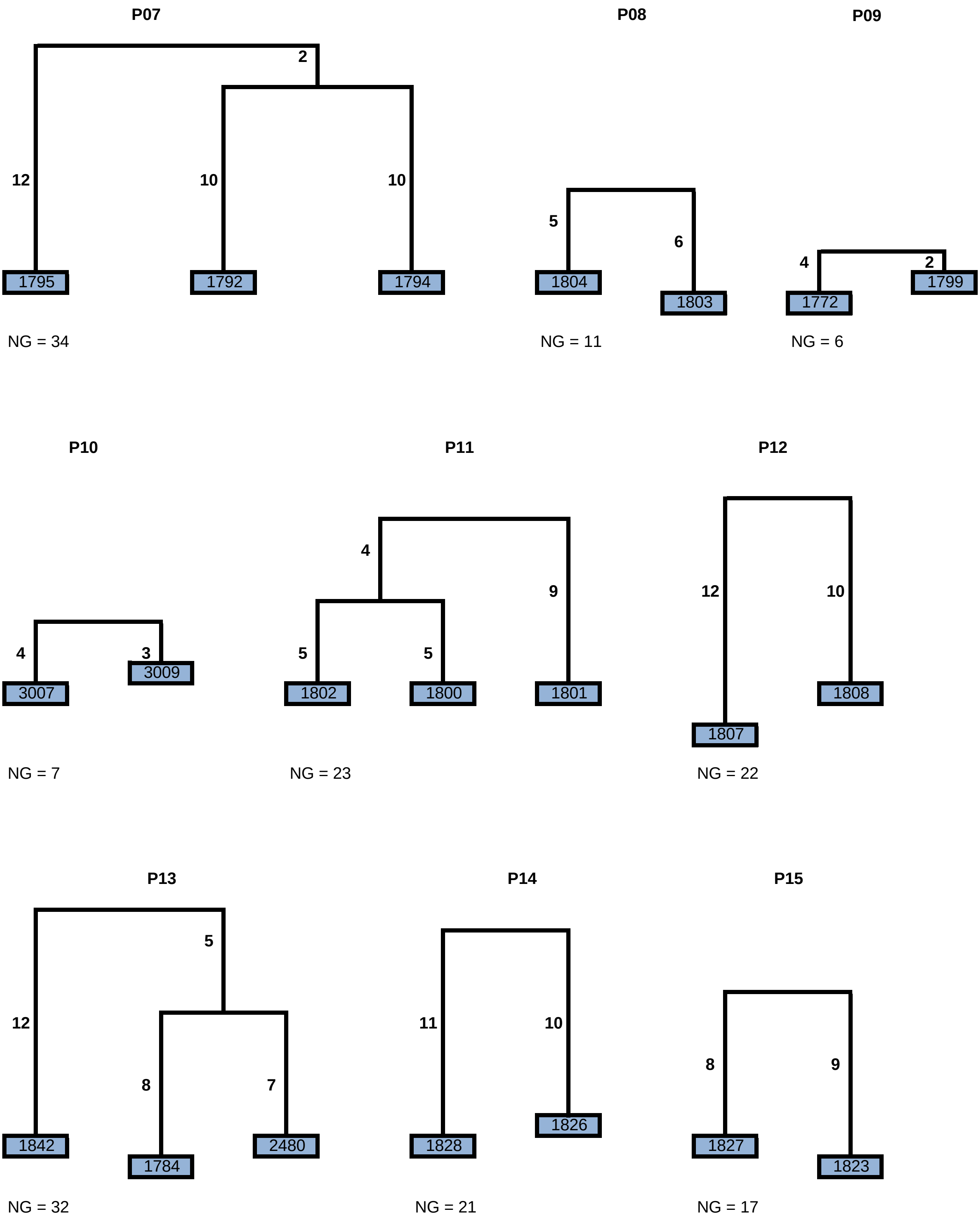
P07
2
12
10
10
1795
1792
1794
NG = 34
P08
5
6
1804
1803
NG = 11
P09
4
2
1772
1799
NG = 6
P10
4
3
3007
3009
NG = 7
P11
4
9
5
5
1802
1800
1801
NG = 23
P12
12
10
1807
1808
NG = 22
P13
5
12
8
7
1842
1784
2480
NG = 32
P14
11
10
1828
1826
NG = 21
P15
8
9
1827
1823
NG = 17

PIEVEQUINTA
(numbers along branches represent the number of generations; NG = total number of generations).

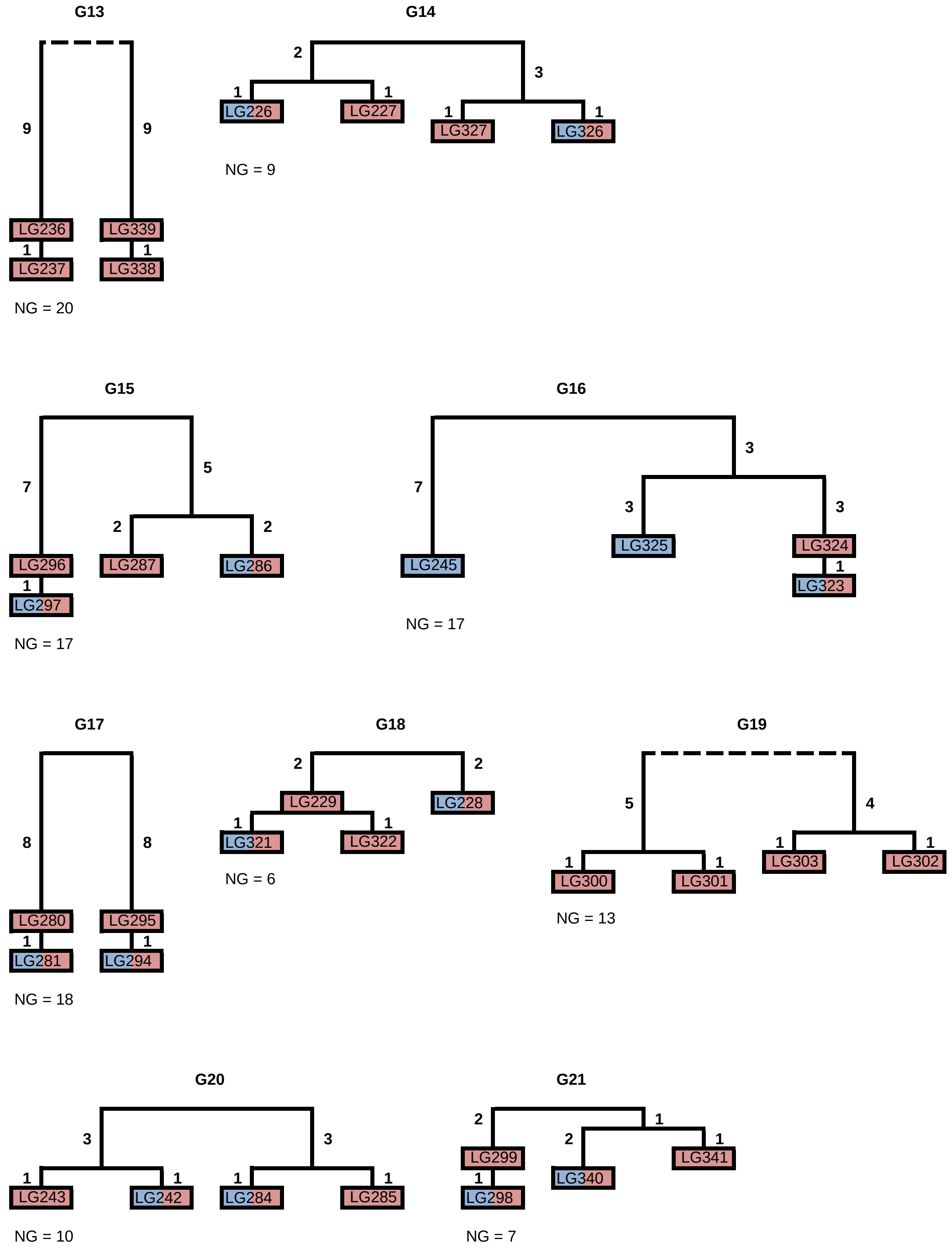

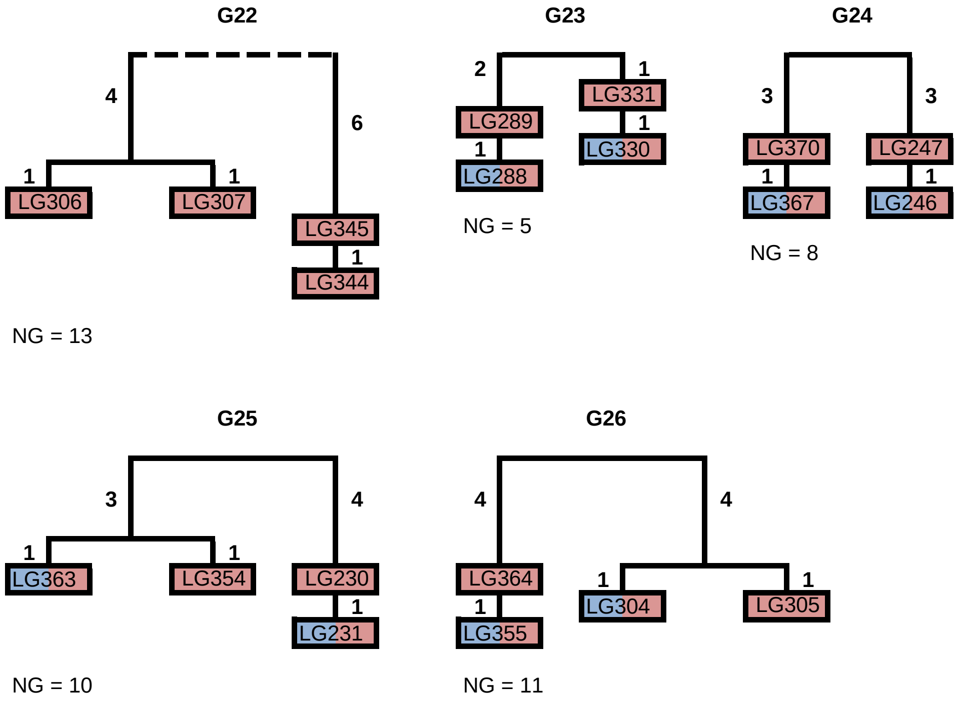

COLOR CODES

- Individuals included in DATASET A
- Individuals included in DATASET B
- Individuals included both in DATASET A and DATASET B
