# Supplementary figures and images for: Mutation Rates and Discriminating Power for 13 Rapidly-Mutating Y-STRs between Related and Unrelated Individuals
Source: PLoS One. 2016 Nov 1;11(11):e0165678. doi: 10.1371/journal.pone.0165678 (PMC5089551; doi:10.1371/journal.pone.0165678)

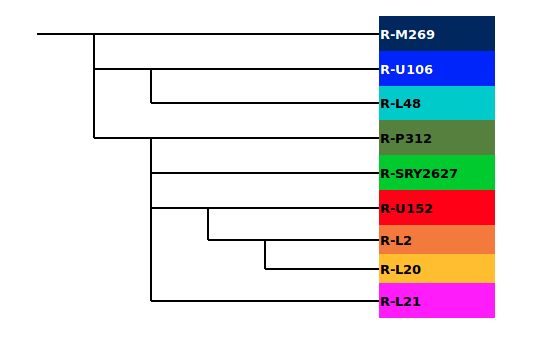

Supplement: S2 Fig — (TIF) [file pone.0165678.s002.tif]
